# Environmental Investigation during Legionellosis Outbreak, Montérégie, Quebec, Canada, 2021

**DOI:** 10.3201/eid2811.220151

**Published:** 2022-11

**Authors:** Laura Atikessé, Nabila Kadaoui, Vincent Lavallée, Éric Levac, Marie St-Amour, François Milord

**Affiliations:** Centre intégré de santé et de services sociaux de la Montérégie-Centre, Longueuil, Quebec, Canada (L. Atikessé, N. Kadaoui, V. Lavallée, É. Levac, M. St-Amour, F. Milord);; Université de Sherbrooke, Longueuil (N. Kadaoui, É. Levac, M. St-Amour, F. Milord);; Public Health Agency of Canada, Ottawa, Ontario, Canada (V. Lavallée)

**Keywords:** Legionella pneumophila, legionellosis, outbreak, water cooling tower, wind rose, Canada, bacteria

## Abstract

In August 2021, a legionellosis outbreak involving 7 persons occurred within a 500-meter radius in the Montérégie region of Québec, Canada. Near real-time modeling of wind direction along with epidemiologic and environmental investigations identified the possible source. Modeling wind direction could help identify likely *Legionella pneumophila* sources during legionellosis outbreaks.

On August 11, 2021, a third reported legionellosis case in the Montérégie region, Quebec, Canada, triggered an outbreak investigation. Using published guidelines for legionellosis investigations (*1*,*2*), the investigation team sought to find the source of infection and rapidly stop the outbreak (*3*). However, confirmation of environmental sources of *Legionella pneumophila*, the bacteria that causes legionellosis, is not always possible (*4*).

The outbreak comprised 7 identified cases, 5 in men and 2 in women. Case-patient ages were 56–85 years, and all had a positive urinary antigen test for *L. pneumophila* serogroup 1. Patient symptoms began during July 29–August 18, 2021; thus, the incubation period was during July 19–August 16. Five case-patients lived within a 500-meter radius in the same neighborhood but were not otherwise acquainted. The other 2 case-patients visited that same area, 1 during July 28–29, the other on August 1.

Within a 3-km radius of the target area, 5 water cooling towers (numbers 1–5) were in operation in 3 facilities. We collected water samples from the 5 towers during August 12–13 and analyzed samples by PCR and culture (Appendix). We also reviewed results of periodic water sampling conducted on the towers during the previous 12 months; only 1 result was above normal, but it was below Quebec’s threshold of 1 million CFU/L for *L. pneumophila*, which requires owners to shut off ventilation, immediately decontaminate the system, and notify public health authorities. A sample collected on July 21 from cooling tower 1 was in the range of 10,000–100,000 CFU/L for *L. pneumophila*, triggering mandatory regulatory remediation actions, which the owner implemented. Nonetheless, we requested the owner of that cooling tower stop using the ventilator until we obtained further results.

After receiving PCR results for all cooling towers, we also requested a ventilator shutdown for cooling tower 5 on August 14 because of *L. pneumophila* detection. Subsequent information showed cooling tower 5 had no biocide during July 26–August 14; the tower was flushed and decontaminated on August 15 and 16, and we collected a control sample after decontamination. We reviewed control results for cooling towers 1 and 5, then informed the buildings’ managers they could restart the ventilators.

Using published protocols (*5*,*6*), the municipality and a private laboratory took water and swab samples from a rainwater retention pond located near cooling towers 1 and 2 and collected samples in 5 aeration ponds of a sewage treatment plant located near the other 3 cooling towers. All 6 ponds were equipped with aerating fountains, but swab samples were taken from the fountain only in the rainwater retention pond. Cultures identified *L. pneumophila* serogroup 2–15 in samples from 1 aerated pond at the sewage treatment plant, but no intervention was undertaken. Cultures from all other ponds were negative.

Only 2 respiratory specimens could be collected from the 7 hospitalized patients. One clinical specimen was *L. pneumophila*–positive by PCR, but cultures were negative, and sequence-based typing was inconclusive; thus, we could not match human and environmental isolates.

We collaborated with Environment and Climate Change Canada (ECCC) to identify potential *L. pneumophila* sources by examining meteorological conditions during exposure periods. ECCC created atmospheric dispersion models to illustrate wind directions during each case-patient’s exposure periods (Figure). Two case-patients were not residents of the area and only visited the community for a few hours, which enabled us to use the wind data as discriminatory support for our source hypothesis. ECCC’s model showed the most likely sources were cooling towers 1 and 2 and a nearby rainwater retention pond (Appendix Figures 1, 2). Using near real-time modeling, we triaged and prioritized investigation of exposure sources in the south and west because model findings illustrated little likelihood that exposure originated from the north or northeast (Figure). Modeling showed cooling tower 5 was a low probability source, but absence of biocides during the exposure period raises questions.

**Figure:**
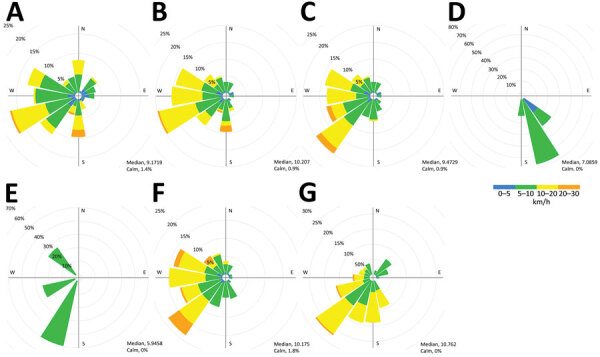
Wind rose profiles during each patient’s exposure period used in environmental investigation for legionellosis outbreak, Montérégie, Quebec, Canada, 2021. A–G) Cases C1–C7. Center crosshairs indicate center of the target area for each case; radii indicate percentage of frequency of winds over a time period, plotted by wind direction, with color bands showing wind speed ranges. Median and calm windspeeds are indicate for exposure times for each case. We calculated wind rose profiles for each of 7 case-patients during the time they were likely exposed. Wind rose profiles were generated by using meteorological data from High Resolution Deterministic Prediction System (HRDPS) modeling. Most case-patients, C1–C3 (panels A–C), C6 (panel G), and C7 (panel F), resided in the outbreak neighborhood; cases C4 (panel D) and C5 (panel E) were only in the area for a few hours, enabling more discriminating assessment of the possible exposures. C, case.

Another legionellosis outbreak investigation in Canada showed that few cases result from exposure within a 3- to 10-km radius of the *L. pneumophila* source (*7*). Nonetheless, we expanded our search to cooling towers within a 10-km radius of the target area (cooling towers 6–11) but found no contributing source (Appendix Figures 1, 2). 

Our environmental investigation included supplementary information from many partners. Because outbreaks have been linked to misting equipment in grocery stores (*8*) and 5 of 7 case-patients shopped at the same grocery store, we took samples from the store’s water system and misting nozzles, but all cultures were negative for *L. pneumophila*. In addition, no tanker trucks were used for cleaning or reducing dust on the roads nor watering flowers in the affected area.

In conclusion, although none of the sampled cooling towers had microbiologic results above the sanitary threshold and we were not able to confirm the source by sequence-type matching, evidence suggests that cooling tower 1 was involved in this legionellosis outbreak. This investigation showed the usefulness of near real-time wind direction modeling, which could help identify likely *L. pneumophila* sources in future outbreaks.

Appendix Additional information on environmental investigation during legionellosis outbreak, Montérégie, Quebec, Canada, 2021.
