# Supplementary material for: Environmental Investigation during Legionellosis Outbreak, Montérégie, Quebec, Canada, 2021
Source: Emerg Infect Dis. 2022 Nov;28(11):2357–60. doi: 10.3201/eid2811.220151 (PMC9622261; doi:10.3201/eid2811.220151)
Supplement: Appendix — Additional information on environmental investigation during legionellosis outbreak, Montérégie, Quebec, Canada, 2021. [file 22-0151-Techapp-s1.pdf]

Article DOI: https://doi.org/10.3201/eid2811.220151

Environmental Investigation during Legionellosis Outbreak, Montérégie, Quebec, Canada, 2021

Appendix

Results from Water Samples Collected from Cooling Towers

We sent samples taken on August 12 and 13 from suspect cooling towers to Centre d'expertise en analyse environnementale du Québec (the Center of Expertise in Environmental Analysis of Quebec) to conduct PCR and culture analyses. Unlike cultures, for which a result is possible after ≈14 days, PCR analyses are available within a short period, 24–48 h. We sent isolates from cultures to the Laboratoire de santé publique du Québec (Quebec public health laboratory) for identification of *Legionella pneumophila* by sequence-based typing.

PCR analyses were inconclusive for cooling towers 2, 3, and 4. The culture showed the presence of *L. pneumophila* for the cooling tower 4 with a low result of 2,500 CFU/L. No close follow-up was done with this cooling tower because the previous and subsequent monthly control samples were under the remediation action level (i.e., <5,000 CFU/L) and the owner did not report any deficiency in his logbook.

Appendix Table. Results of PCR, culture, and *Legionella pneumophila* sequence-based typing analyses from environmental investigation of legionellosis outbreak, Montérégie, Quebec, Canada, 2021*

Cooling tower no., facility	PCR, GU/L	Culture, CFU/L	Sequence-based typing
CT no. 1, facility A	4,700	5,000	ST1
CT no. 2, facility A	<760	<2,500	None
CT no. 3, facility B	Nonquantifiable	<2,500	None
CT no. 4, facility B	Nonquantifiable	2,500	ST1
CT no. 5, facility C	<190,000†	6,700	ST3023

*Samples were collected from cooling towers located on facilities in a 3-km area. CFU, colony forming units; CT, cooling tower; GU, genomic copies; ST, sequence type.
†*L. pneumophila* detected, but nonquantifiable because of presence of suspended matter and inhibitors.

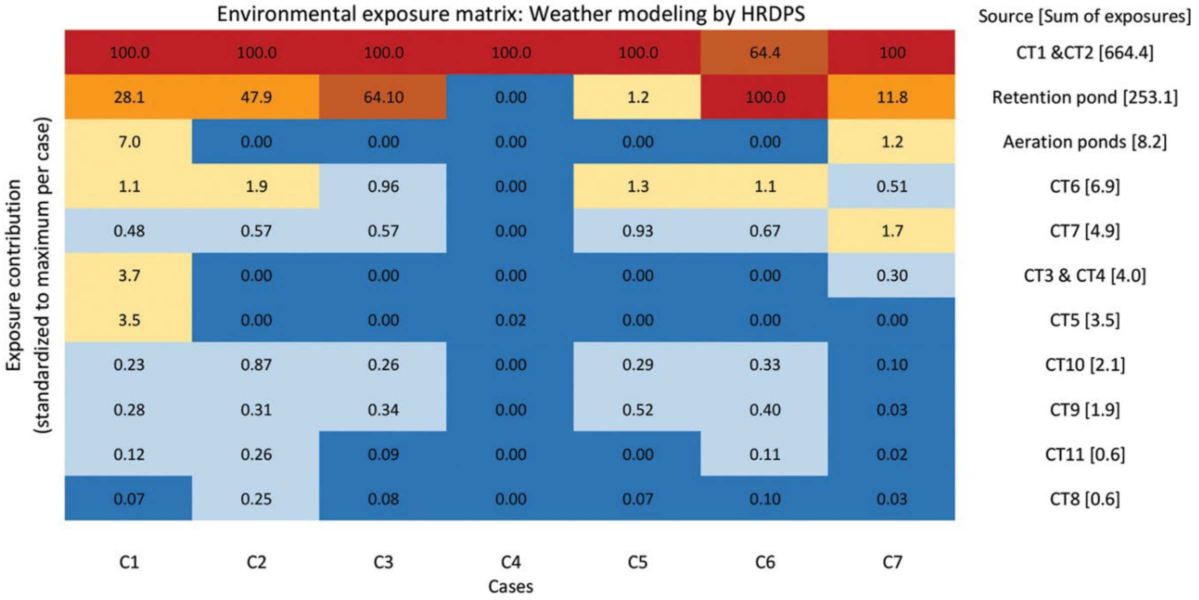

Appendix Figure 1. Gaussian atmospheric dispersion modeling of *Legionella pneumophila* exposures noted during environmental investigation of legionellosis outbreak, Montérégie, Quebec, Canada, 2021. Using High Resolution Deterministic Prediction System (HRDPS), our partners at Environment and Climate Change Canada (ECCC) created a Gaussian atmospheric dispersion model for each of the suspected environmental sources of *Legionella pneumophila* in this outbreak. Using this model, ECCC produced an environmental exposure matrix for each case at the case-patient’s primary exposure site and during their exposure periods. Numerals in each box indicate the relative percentage contribution or exposure each site is estimated to contribute, standardized to the highest contributing source for each case (index = 100). *L. pneumophila* sources are ordered in ascending order of the sum of the exposure estimates indicating that cooling towers at facility A (CT1 & CT2) are the most likely source, then a nearby rainwater retention pond. The Gaussian model suggests notable decreases in likelihood for the subsequent sources as determined by the sum of the exposures. CT6–CT11 are located in a 3- to 10-km radius of the target area. CT, cooling tower.

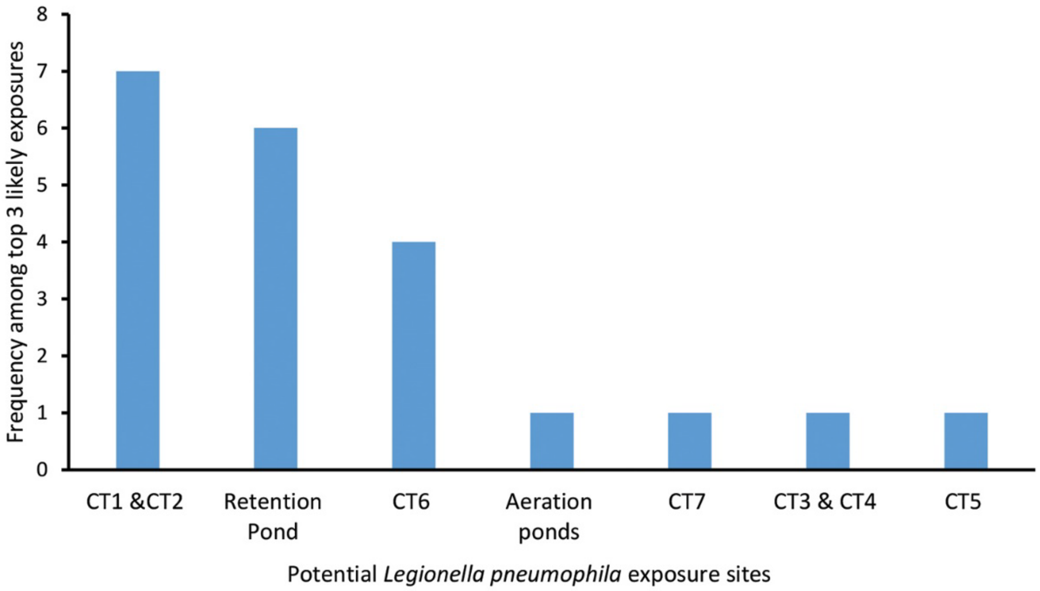

Appendix Figure 2. Compilation of most frequently contributive sources from HRDPS Gaussian atmospheric dispersion modeling during environmental investigation of legionellosis outbreak, Montérégie, Quebec, Canada, 2021. We ranked the frequency of sources for top 3 sources most likely to contribute to *Legionella pneumophila* exposure as determined by the environmental exposure matrix. The results illustrate that the facility A (CT1 and CT2) was among the top 3 contributive sources for all 7 cases, as was a nearby rainwater retention pond, which scored among the top 3 sources for 6 of the 7 cases. CT, cooling tower.
